# Dual-Enzyme Crosslinking and Post-polymerization for Printing of Polysaccharide-Polymer Hydrogel

**DOI:** 10.3389/fchem.2020.00036

**Published:** 2020-01-30

**Authors:** Saiji Shen, Jiayin Shen, Hongdou Shen, Chu Wu, Ping Chen, Qigang Wang

**Affiliations:** School of Chemical Science and Engineering, School of Life Science and Technology, Tongji University, Shanghai, China

**Keywords:** enzymatic crosslinking, enzymatic polymerization, composite hydrogel, 3D printing, adjustable-strength

## Abstract

Polymer hydrogels are ideal bioprinting scaffolds for cell-loading and tissue engineering due to their extracellular-matrix-like structure. However, polymer hydrogels that are easily printed tend to have poor strength and fragile properties. The gradually polymerized reinforcement after hydrogel printing is a good method to solve the contradiction between conveniently printed and high mechanical strength requirement. Here, a new succinct approach has been developed to fabricate the printable composite hydrogels with tunable strength. We employed the HRP@GOx dual enzyme system to initiate the immediate crosslinking of chondroitin sulfate grafted with tyrosine and the gradual polymerization of monomers to form the composite hydrogels. The detailed two-step gelation mechanism was confirmed by the Fluorescence spectroscopy, Electron paramagnetic resonance spectroscopy and Gel permeation chromatography, respectively. The final composite hydrogel combines the merits of enzymatic crosslinking hydrogels and polymerized hydrogels to achieve adjustable mechanical strength and facile printing performance. The dual-enzyme regulated polymer composite hydrogels are the promising bioscaffolds as organoid, implanted materials, and other biomedical applications.

## Introduction

Hydrogels with three-dimensional networks have received wide attention due to their successful applications in tissue engineering (Yue et al., 2015; Mohamad et al., 2018; Qu et al., 2018, 2019; Edri et al., 2019; Feng et al., 2019), biocatalysis (Diaz et al., 2010), biosensor (Wang et al., 2019), cell culture (Lou et al., 2017; Luo et al., 2019), drug delivery (Gao et al., 2018; Xu et al., 2019), bio-medicines (Liow et al., 2017), wound healing (Ahmed et al., 2017), and 3D printing (Censi et al., 2011; Pataky et al., 2012; Malda et al., 2013; Li et al., 2015; Loebel et al., 2017; Lin et al., 2019). In general, hydrogels formed by natural polysaccharides are suitable for 3D printing and encapsulating biomolecules. However, they generally have poor elasticity and weak mechanical properties. The covalently bonded crosslinked network (polymeric hydrogel) is elastically deformable, and still maintains strong mechanical properties (Sun et al., 2012; Zhang X. N. et al., 2018). However, they generally lack a suitable porous structure to diffuse biomolecules, and it is difficult to obtain a good viscidity window for extruded 3D printing, which are important factors in tissue-repair and 3D printing. An effective way to ensure these two advantages is to use a polysaccharide hydrogel in combination with a polymeric material.

Free radical polymerization (Hume et al., 2011) is the most common technique used to prepare polymer hydrogels. Utilization of enzyme to catalyzes the polymerization of monomers is a suitable method for constructing a biomimetic material, due to enzymatic polymerization has the advantages of high efficiency, high specificity, controllable activity, mild reaction conditions and no toxic residues compared with traditional polymerization. As early as 1950, researchers used xanthine oxidase for catalytic reactions (Kalckar et al., 1950). Our group and other researchers have explored several oxidoreductases to catalyze electron transfer reactions and produce free radicals that can initiate the formation of polymer hydrogels (Wei et al., 2016; Zhang et al., 2017; Zhang Q. et al., 2018). For the typical HRP/laccase initiation system, reductive acetylacetone is catalytically oxidized to form carbon radicals and initiate polymerization by hydrogen peroxide or oxygen, respectively (Tsujimoto et al., 2001; Kohri et al., 2012). Enzyme-catalyzed tyrosine-modified polysaccharides and proteins are a very effective strategy for the preparation of injectable and printable hydrogels, which are a free radical-induced cross-linking process. We note that the HRP/GOx/Glucose system can also initiate *in situ* free radical polymerization of small molecular monomers beyond the radical-crosslinking of tyrosine. This may provide an inspiring approach for designing the printable composite hydrogel with tunable mechanical strength.

Despite the success in the design of composite hydrogels, the use of hard hydrogels for 3D printing to generate complex structures remains a huge challenge. Therefore, we report a concise dual enzyme HRP/GOx-mediated redox initiation to achieve crosslinking of modified polysaccharides and further co-polymerized with monomers to achieve high toughness composite hydrogel. The crosslinkable polysaccharide hydrogel with lower mechanical strength can be printed in a 3D structure due to the printable viscidity window and fast crosslinking. Under the action of time, the dual-enzyme and tyrosine of the polysaccharide system further initiate polymerization of the various monomers. The co-polymerization with the double bond grafted polysaccharide gradually increases the toughness of the hydrogel to obtain a composite hydrogel which has good mechanical properties.

**Figure 1:**
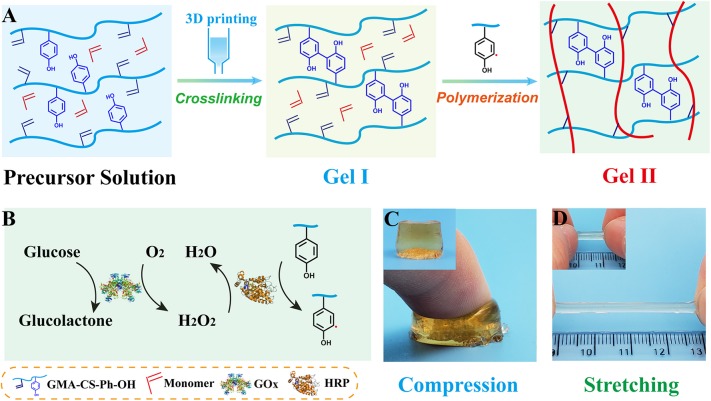
**(A)** Schematic of the preparation of Gel I and Gel II; **(B)** Enzymatic reaction illustration of HRP@GOx system; **(C)** Optical image of Gel II under compression; **(D)** Optical image of Gel II under stretching [Gel II in **(C,D)** have different diameter, which cause different colors to be reflected under the same shooting conditions].

There are two processes in designing of composite hydrogels (Figure 1A): (i) crosslinkable hydrogel (Gel I) and (ii) polymeric/crosslinked hydrogel (Gel II). The entire hydrogel precursor solution consists of horseradish peroxidase (HRP), glucose oxidase (GOx), glucose, grafted tyrosine and double bond chondroitin sulfate (GMA-CS-Ph-OH), acrylamide monomers and deionized water. First, GOx catalyzes the oxidation of glucose to gluconic acid and H_2_O_2_, then HRP and H_2_O_2_ oxidize tyrosine-modified chondroitin sulfate to immediately form Gel I. Then, tyrosine form α-carbon radical under the oxidation of the HRP@GOx system to gradually initiate polymerization of the monomer and crosslink with the polysaccharide grafting double bond to form Gel II. The α-carbon radicals from tyrosine molecules were detected by electron paramagnetic resonance (EPR) measurements (Figure 6B).

## Materials and Methods

### Materials

Acrylamide (AAm), tyramine hydrochloride were purchased from Aladdin Industrial Corporation. N, N-Dimethyl Acrylamide (DMAA), N-isopropyl acrylamide (NIPAM) were purchased from Tokyo Chemical Industry Co., Ltd. Poly (ethylene glycol) methacrylate (PEGMA) was purchased from Sigma-Aldrich (Shanghai) Trading Co., Ltd. Glycidyl methacrylate (GMA), N-hydroxysuccinimide (NHS), 1-(3-Dimethylaminopropyl)-3-ethylcarbodiimide hydrochloride (EDC-HCl), and deuteroxide (D_2_O) were purchased from Energy Chemical. Hydrochloric acid (HCl), D-(+)-Glucose were purchased from Sinopharm Chemical Reagent Co., Ltd. Chondroitin sulfate (CS, Molecular weight: 5 × 10^4^ KD), Horseradish peroxidase (HRP, ≥300 U/mg), Glucose oxidase (GOx, 239 U/mg) and dialysis bag were purchased from Shanghai Baoman Biotechnology Co., Ltd. Deionized water was used to prepare the solution throughout the experiments unless otherwise stated.

### Methods

#### Synthesis of GMA-CS

2.00 g of chondroitin sulfate was added to 50 ml of deionized water, stirred until the chondroitin sulfate was completely dissolved in room temperature, and pH was adjusted to 3.5 with HCl. Then, 521.2 mg of GMA was added, and the system was stirred at 50°C for 24 h. After completion, it was dialyzed in deionized water for 3 days with a dialysis bag which has a molecular weight of 3,500 to remove impurities, and the dialyzed liquid was placed in a freeze dryer to remove water to obtain GMA-CS.

#### Synthesis of GMA-CS-Ph-OH

2.00 g of GMA-CS was added to 50 ml of deionized water, stirred until the GMA-CS was completely dissolved in room temperature. Then, 695.4 mg of tyramine hydrochloride, 1151.6 mg of EDC-HCl and 691.4 mg NHS were added, and the reaction was stirred at room temperature for 12 h. After completion, it was dialyzed in deionized water for 3 days with a dialysis bag which has a molecular weight of 3,500 to remove impurities, and the dialyzed liquid was placed in a freeze dryer to remove water to obtain tyrosine modified GMA-CS (GMA-CS-Ph-OH).

#### Preparation of Functionalized Chondroitin Sulfate Crosslinking Hydrogel (Gel I) and Composite Hydrogel (Gel II)

GMA-CS-Ph-OH, monomers, HRP (20 mg/ml), and GOx (20 mg/ml) were completely dissolved in deionized water at room temperature to prepare a precursor solution. Subsequently, 100 mM Glucose was quickly added to the precursor solution and stirred uniformly. After a period of standing, the GMA-CS-Ph-OH in the precursor solution immediately formed a crosslinking hydrogel (Gel I) under the oxidation of the HRP@GOx system. After forming the crosslinking hydrogel, the HRP@GOx system in Gel I will continue to oxidize tyrosine, causing tyrosine to generate α-carbon free radicals, gradually initiating polymerization of the monomer to form composite hydrogel (Gel II).

#### Characterization

The mechanical properties of the hydrogel were characterized via utilizing an electronic universal testing machine. ^1^H-NMR was used to characterize conversion rates of GMA-CS-Ph-OH and monomers. The network structure inside the hydrogel was characterized by using Scanning Electron Microscopy (SEM). The frequency sweep tests of the hydrogel were characterized via using a rheometer (Rheometer). The tyrosine/di-tyrosine structure of precursor solution/Gel I were characterized by utilizing F-7000 FL Spectrophotometer. The detection of carbon free radicals were performed via using the Bruker Electron Paramagnetic Resonance (EPR). The degree of polymerization of the Gel I and Gel II were tested by using Gel Permeation Chromatography (GPC). The crosslinking hydrogel (Gel I) was printed via GeSim bioscaffold 3.2 3D printer.

## Results and Discussion

The preparation of the composite hydrogel is straightforward and requires no additional initiator. First, GMA-CS-Ph-OH was oxidized under the catalysis of dual enzyme catalytic system HRP@GOx to form α-carbon radicals (Figure 1B). Subsequently, a GMA-CS-Ph-OH hydrogel with three-dimensional network structure (Gel I, Figure 1A) was immediately formed via crosslinking reaction. Due to the strong steric-hindrance effect of crosslinked GMA-CS-Ph-OH, GMA-CS-Ph-OH with α-carbon radicals presented in the system won't occur crosslinking reaction after Gel I formation. Finally, the acrylamide monomers were polymerized under the initiation of α-carbon radicals, and gradually formed composite hydrogel (Gel II) with a semi-interpenetrating network structure with Gel I (Figure 1A). Therefore, the composite hydrogel can be formed by simply mixing the reaction solution and maintaining it at room temperature.

**Figure 2:**
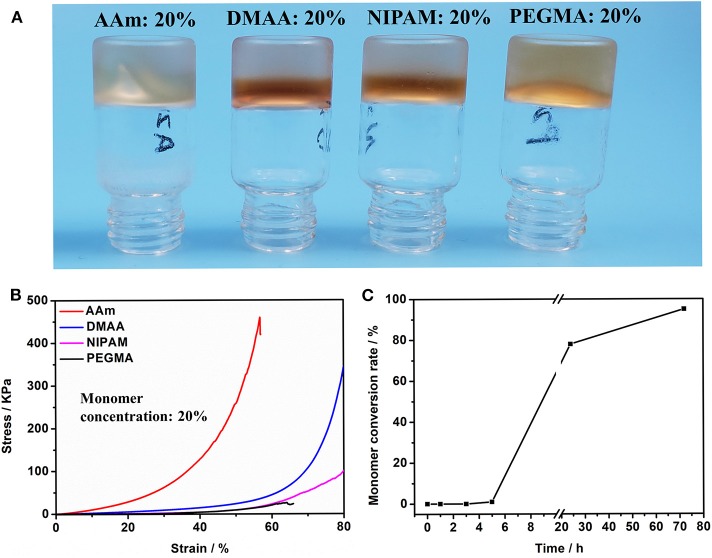
**(A)** Optical image of Gel II composed of different monomers (including AAm, DMAA, NIPAM, and PEGMA); **(B)** Compressive tests of Gel II composed of different monomers (including AAm, DMAA, NIPAM, and PEGMA). **(C)** The conversion of AAm in Gel II is calculated using the ^1^H-NMR spectra.

Subsequently, we investigated the polymerization reaction of a number of acrylamide monomers catalyzed by HRP@GOx. As shown in Figure 2A, AAm, DMAA, NIPAM, and PEGMA can be all polymerized under the catalysis of HRP@GOx, and form Gel II with a semi-interpenetrating network structure with Gel I. Its mechanical properties are determined by the intrinsic properties of the monomer, such as the molecular structure (Figure 2B). The reason for this phenomenon is that GMA-CS-Ph-OH generates α-carbon radicals under the catalysis of HRP@GOx, and then non-selective α-carbon radical can initiate *in situ* polymerization of acrylamide monomers without the need for additional initiators.

**Figure 3:**
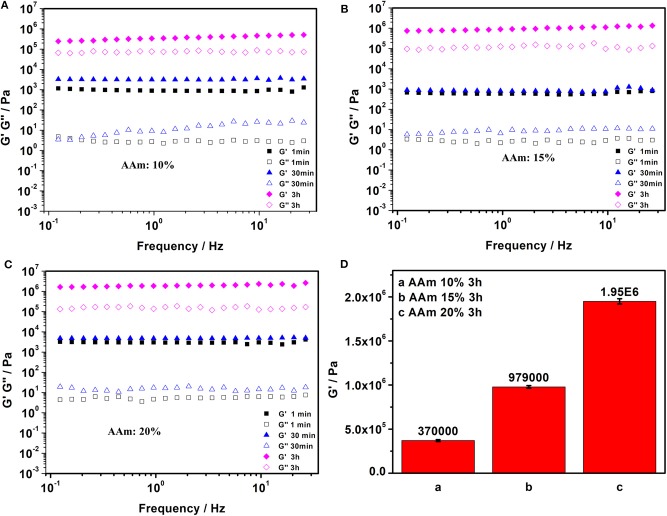
Frequency sweep tests of Gel II contains **(A)** 10 wt% AAm, **(B)** 15 wt% AAm, and **(C)** 20 wt% AAm when the reaction time were 1 min, 30 min, and 3h; **(D)** The value of G' in Frequency sweep tests of Gel II contains 10 wt% AAm, 15 wt% AAm, 20 wt% AAm, respectively, when the reaction time was 3 h. Each bar represents the mean (*n* = 22) ± SD.

**Figure 4:**
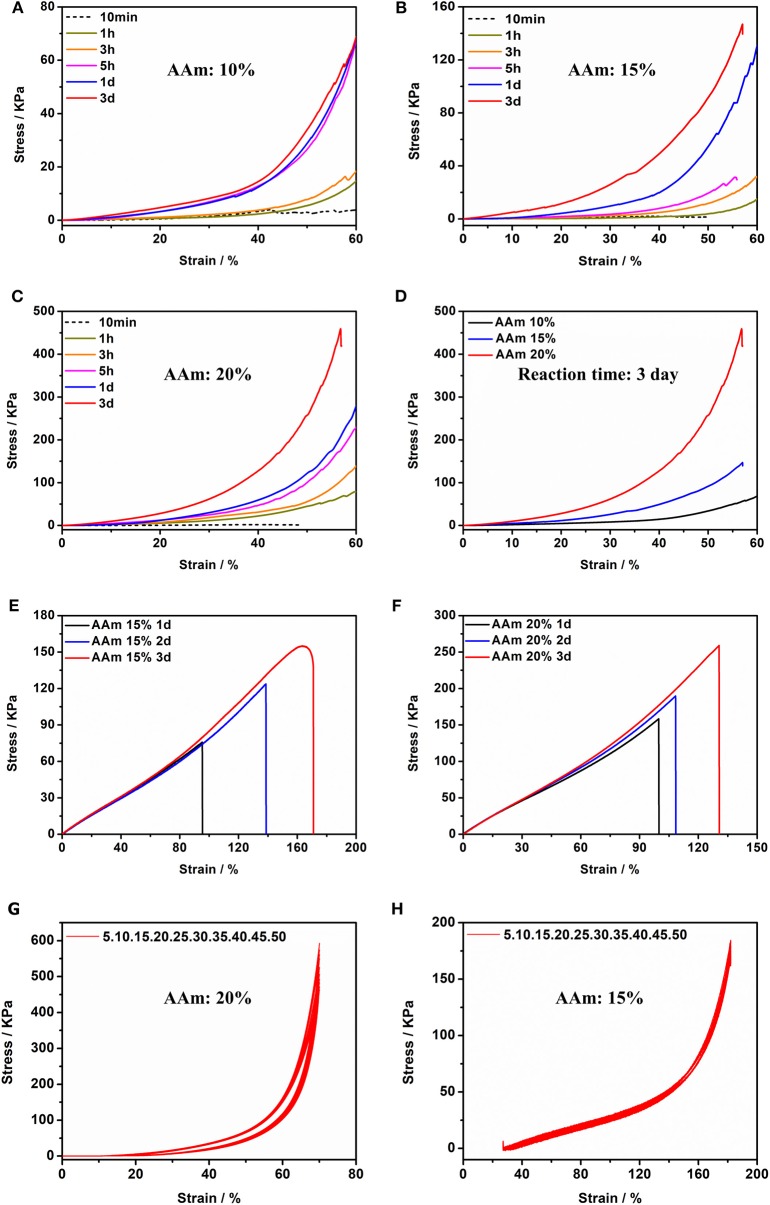
Compression tests of Gel II contains **(A)** 10 wt% AAm, **(B)** 15 wt% AAm and **(C)** 20 wt% AAm when the reaction time were 10 min, 1, 3, 5 h, 1 d, and 3 d **(D)** Compression tests of Gel II contains 10, 15, and 20 wt% AAm, respectively, when the reaction time was 3 d; Tensile test of Gel II contains **(E)** 15 wt% and **(F)** 20 wt% AAm when the reaction time was 1, 2, and 3 d; **(G)** Cyclic compression test of Gel II contains 20 wt% AAm; **(H)** Cyclic tensile test of Gel II contains 15 wt% AAm.

The acrylamide (AAm) was experiment as a template molecule. The gelation process was monitored via ^1^H-NMR (25°C). After 5 h, the intensity of the characteristic peak of the double group of AAm began to decrease significantly. After 3 days, the double group of AAm were saturated quantitatively (Figure 2C), which corresponded to the formation of Gel II, whereas the gelator conversion test showed that 94.9 ± 1.2% (mean ± SD) of AAm participated in the polymerization reaction. In order to evaluate the effect of quantity of AAm in the crosslinking process, a series of composite hydrogels containing different amounts of AAm (10, 15, 20 wt%) were prepared for comparison of mechanical properties. Gel II containing different concentrations of AAm all reached optimal mechanical properties within 3 days of reaction. Figures 3A–D revealed the results of frequency-dependent sweep measurements at a constant strain of 0.03%. The values for both storage modulus (G′) and loss modulus (G″) increased gradually with an increase in the amount of AAm. When the amount of AAm was 20 wt%, the G' of Gel II reached 1.95×10^6^ Pa. the G' of Gel II was 0.37×10^6^ Pa when the amount of AAm is 10 wt%. Whether the amount of AAm is 10 or 20 wt%, the values of storage modulus (G′) always much higher than loss modulus (G″), which confirmed inherent high elastic properties of Gel II. In the compression test, the fracture stress and the elastic modulus also increased as the amount of AAm increased (Figures 4A–D). When the amount of AAm is 15 wt%, the tensile properties were optimal (Figures 4E,F) (Gel II can be stretched 2.7 and 2.3 times, respectively, with 15 and 20 wt% AAm, and cannot be stretched with 10 wt% AAm). According to the above discussion, Gel II with different mechanical properties can be obtained by change the amount of AAm. The compression cycle and the tensile cycle test were, respectively, performed on Gel II contains 20 and 15 wt% AAm. Even after 50 cycles, the compression and tensile curves were almost coincident, indicating that Gel II formed under the catalysis of HRP@GOx without additional initiators has excellent stability (Figures 4G,H).

**Figure 5:**
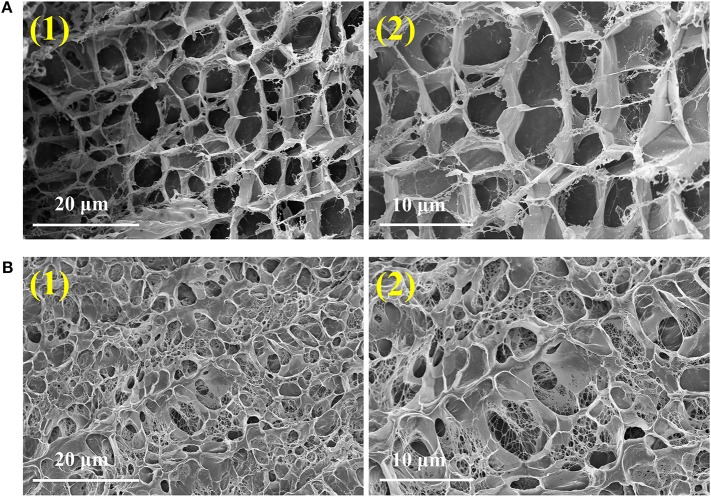
SEM images of Gel I **(A)** and Gel II **(B)**.

Scanning Electron Microscopy (SEM, Figure 5) confirmed that Gel II had a similar porous network structure as Gel I, but with smaller pore size and closer network structure. The SEM image of Gel I (Figure 5A) exhibited entangled irregular fibers structure, which was the matrix of Gel II (Figure 5B) had a different morphology with fibers that were tightly crosslinked to each other and intertwined with Gel I. It is proved that the density of nanofibers in Gel II is higher than that of the Gel I network, which proves that the crosslinking process of GMA-CS-Ph-OH promotes the formation of Gel II.

**Figure 6:**
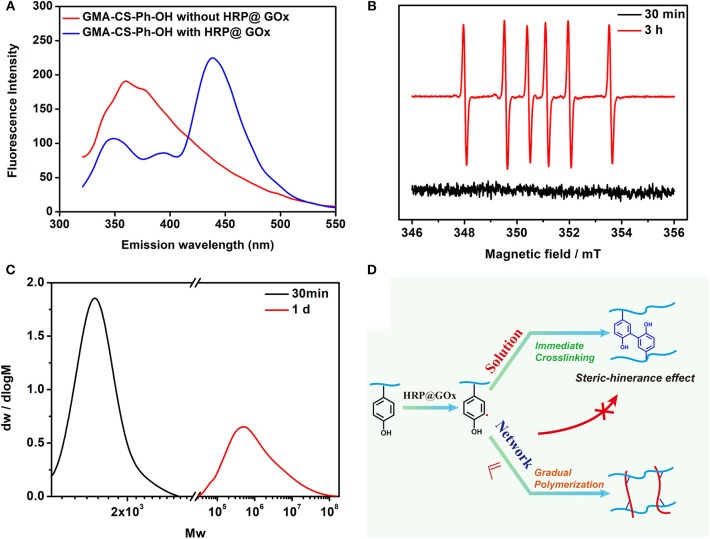
**(A)** Fluorescence Spectroscopy spectrum of GMA-CS-Ph-OH with and without HRP@GOx; **(B)** EPR spectrum of the DMPO radical adduct formed in HRP@GOx, GMA-CS-Ph-OH, and AAm reaction system at 30 min (black line) and 3 h (red line); **(C)** The GPC spectrum of Gel I (black line) and Gel II (red line); **(D)** Mechanism illustration of catalytic oxidation of tyrosine via HRP@GOx system.

Fluorescence Spectroscopy, Electron paramagnetic resonance (EPR) spectroscopy, and Gel Permeation Chromatography (GPC) were performed to verify the gelation mechanism. When the excitation wavelength is 300 nm, Tyrosine has a characteristic fluorescence emission peak at 360 nm, while di-tyrosine has a characteristic fluorescence emission peak at 425 nm. Therefore, fluorescence spectroscopy was used to characterize the reaction process. As shown in Figure 6A, GMA-CS-Ph-OH exhibited a characteristic fluorescence emission peak of tyrosine at ca. 360 nm. When the HRP@GOx catalytic system was presented, not only the characteristic fluorescence emission peak of di-tyrosine occurs at 438 nm, but also the characteristic fluorescence emission peak belonging to tyrosine appears at 348 nm. This may be attributed to the steric-hindrance effect of the GMA-CS-Ph-OH crosslinking reaction, which results in the presence of a portion of unbound GMA-CS-Ph-OH in the system. The characteristic fluorescence emission peaks of tyrosine and di-tyrosine were both shifted, which may be attributed to the influence of the molecular structure of the enzyme. EPR was utilized to detect radicals in the system at different reaction times. Dimethyl pyridine N-oxide (DMPO) was employed to trap the unstable and short-lifetime free radicals to form a long-lived nitroxide for EPR characterization. As shown in Figure 6B (black line), no carbon radicals were detected at the reaction for 30 min, which was belonged to the fact that the GMA-CS-Ph-OH molecules were oxidized to immediately form a dimer via the combination of α-carbon radicals. When the reaction is carried out for 3 h, the EPR spectrum of the reacted system demonstrated a sextet signal (red line) with a *g* value of 2.005, *AN* = 1.67 mT, *AH* = 2.38 mT, which were coincident with the values of a DMPO trapped carbon centered radical (Buettner, 1987), it is attributed to the completion of the crosslinking reaction of GMA-CS-Ph-OH, and remaining carbon radicals in the reaction system will initiate the polymerization of AAm. The EPR results confirmed our hypothesis that there is a process of gradual polymerization of AAm after GMA-CS-Ph-OH immediate crosslinking. The molecular weight distribution of the Gel I and Gel II formed after crosslinking and polymerization were measured by GPC (Figure 6C). The number-average molecular weight (*Mn*) of Gel I and Gel II were 1.67 × 10^3^ and 1.63 × 10^6^, which proves the polymerization of AAm in polysaccharide network. Based on the above results, the gelation mechanism is proposed: as shown in Figure 6D, tyrosine of GMA-CS-Ph-OH generates α-carbon radicals under catalytic oxidation of the HRP@GOx system. In solution environment, α-carbon radicals tend to react with each other to initiate crosslinking reaction of the GMA-CS-Ph-OH. When GMA-CS-Ph-OH was crosslinked and then obtained a highly viscous network structure, α-carbon radicals were difficult to react with each other due to steric-hindrance effect, resulting in α-carbon radicals tending to induce polymerization of acrylamide monomers.

**Figure 7:**
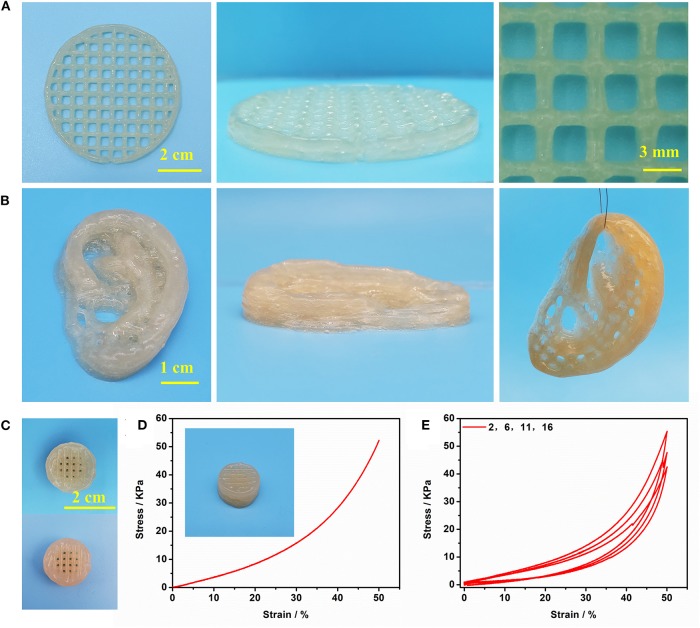
**(A)** The printed shape “button” with several layers; **(B)** The printed shape “ears” with several layers; **(C)** The printed 3D shape “button” (up) and enhanced 3D shape “button” (bottom); **(D)** Compressive test of enhanced 3D shape “button”; **(E)** cyclic compression test of enhanced 3D shape “button”.

Gel II can retain its shape even under compression and tensile relative to the weaker Gel I. As shown in Figure 4C, Gel II can resist over 57% compression and has an 86.73 KPa Young's modulus, whereas Gel I only has a 3.29 KPa Young's modulus. Furthermore, the cylindrical composite hydrogel has well compression performance (Figure 1C) and could be stretched to about 4 times its initial length without collapse (Figure 1D). Because of these properties (such as *in situ* formation and enhanced mechanical properties of Gel II), the precursor solution was used for 3D printing. First, we imported the designed 3D model into the printer, selected the appropriate printing pressure and speed, and then quickly injected the configured precursor solution into the print cylinder for printing. The shapes of “button” (Figure 7A) and “ears” (Figure 7B) with several layers were 3D printed. The printed 3D model was statically cured (Figure 7C) and then subjected to compression test (Figure 7D) and cyclic compression test (Figure 7E), and the 3D model exhibited greater compression performance even after cyclic compression 16 times. This result demonstrates that the composite hydrogels could be used as novel printable scaffold materials with enhanced mechanical properties.

## Conclusion

In summary, we have developed a new strategy for the mild preparation of polymer composite hydrogels for 3D printing via using the dual enzyme HRP@GOx systems. The preparation process of polysaccharide-polymer composite hydrogel comprises two steps: (1) GMA-CS-Ph-OH is immediately crosslinked to form a polysaccharide hydrogel with weak mechanical strength but printable; (2) The gradual polymerization form composite hydrogel of adjustable strength after immediate crosslinking. The monomer conversion of the composite hydrogel was detected to be 95% via ^1^H-NMR. Composite hydrogels have adjustable strength from 3.29 to 86.73 KPa along with various type and concentration of monomers. The mechanism analyses confirmed the immediate cross-linking at diluted solution and gradually polymerized reinforcement within viscous polysaccharide network. Therefore, our polymer composite hydrogels have denser pore and nanoscale network relative to only polysaccharide hydrogels. With excellent biocompatible and mechanically adjustable abilities, the composite hydrogel is particularly interesting for 3D printing to fabricate precision structures for tissue repairing and tissue engineering.

## Data Availability Statement

The datasets generated for this study are available on request to the corresponding author.

## Author Contributions

SS and QW conceived the study. SS, JS, HS, CW, PC, and QW had input in the experimental design. SS and QW analyzed the data. SS drafted the manuscript with support from QW and PC. All authors read, commented on, and approved the paper.

### Conflict of Interest

The authors declare that the research was conducted in the absence of any commercial or financial relationships that could be construed as a potential conflict of interest.
